# Gender Differences in Community-acquired Meningitis in Adults: Clinical Presentations and Prognostic Factors

**Published:** 2016-04-08

**Authors:** Lavanya Dharmarajan, Lucrecia Salazar, Rodrigo Hasbun

**Affiliations:** Department of Internal Medicine, University of Texas Health Science Center in Houston, USA

**Keywords:** Gender differences, Meningitis, Community-acquired

## Abstract

Community-acquired meningitis is a serious disease that is associated with high morbidity and mortality. The purpose of this study was to investigate the gender differences involved with the clinical presentations of and prognostic factors for this disease. We conducted a retrospective study of 619 adults diagnosed with community-acquired meningitis in Houston, Texas, who were hospitalized between 2005 and 2010. Patients were categorized as male or female. Those who were evaluated to have a Glasgow Outcome Scale score of four or less were classified to have an adverse clinical outcome. Males consisted of 47.2% (292/619) of the total cohort, and more often presented with coexisting medical conditions, fever, abnormal microbiology results, and abnormalities on head computed tomography. Females more often presented with nuchal rigidity. On logistic regression, fever, CSF glucose <45 mg/dL, and an abnormal neurological examination were predictors of an adverse outcome in male patients, while age greater than 60 years and an abnormal neurological examination were associated with a poor prognosis in female patients. Thus, community-acquired meningitis in males differs significantly from females in regards to comorbidities, presenting symptoms and signs, abnormal laboratory and imaging analysis, and predictors of adverse clinical outcomes.

## Introduction

Community-acquired meningitis can be caused by several treatable and untreatable infectious etiologies (e.g., bacterial, viral, and fungal infections), but most commonly the etiology is unknown [1,2]. Studies show that risk factors for bacterial meningitis include age, immunosuppression, genetic susceptibility, and anatomical defects [3]. Mortality and morbidity for bacterial meningitis is high, with risk factors for a poor outcome including systemic compromise and a low level of consciousness [4]. Outcome largely depends on rapid initiation of an effective empiric treatment, adjusting for age, systemic symptoms, and antimicrobial resistance [5].

While there is some understanding of the effect of age and other risk factors on susceptibility to community-acquired meningitis, there are no studies exploring gender differences in community-acquired meningitis. The purpose of this study is to investigate gender differences in clinical presentation, laboratory and imaging results, etiologies and prognostic factors in male and female patients with community-acquired meningitis.

## Methods

### Case definition

This is a sub-study of another community-acquired meningitis study [2]. Each adult patient (older than 17 years) enrolled in the study had community-acquired symptoms of meningitis (such as headache, stiff neck, fever, focal neurological deficits, or altered mental status) and a cerebrospinal fluid (CSF) white cell count of greater than 5 cells/mm^3^. These patients presented to an emergency department at a Houston-area hospital between 2005 and 2010. The University of Texas Health Science Center in Houston Committee for the Protection of Human Subjects and the Memorial Hermann Hospital Research Review Committee approved this study.

### Data collection, laboratory testing and definition of diagnostic outcomes

Patients’ baseline characteristics were recorded when they were seen in the emergency department. Data on sociodemographic factors, comorbid conditions (as measured by the Charlson comorbidity scale [6]), immunodeficiency risk factors, clinical findings (including neurological exam findings and evaluation on the Glasgow coma scale [7]), laboratory values and management decisions were also collected. Brain imaging, including head computerized tomography (CT) scans and magnetic resonance imaging (MRI), were read by board-certified neuroradiology faculty and were deemed abnormal based on the presence of intracranial parenchymal abnormalities.

The different etiologies of community-acquired meningitis that the patients presented with were categorized into one of the following groups: a) unknown cause; b) untreatable cause; c) treatable but not urgent cause; d) urgent treatable causes [1,2]. The endpoint for this study was the presence of an adverse clinical outcome, which was evaluated at the time of patient discharge using the Glasgow outcome scale, as described in previous studies [2,4]. In this study, an adverse clinical outcome constituted a Glasgow outcome score of less than four.

### Statistical analysis

Patient characteristics that could clinically have an association with an adverse outcome were evaluated by bivariate analysis with Fisher-exact test, Chi square, and Student t-test. Significant variables on bivariate analysis (p<0.05) were entered into a logistic regression model with bootstrapping analysis for validity [8]. No more than one variable was entered per six outcome events [9]. All statistical analysis were done with IBM^®^ SPPS^®^ version 21.

## Results

### Cohort assembly

In assembling the cohort, 727 patients with community-acquired meningitis were screened, and 108 patients were excluded for a variety of reasons, as described in the original study [2]. After the exclusion criteria, 619 patients were enrolled and divided into male (n=292) and female (n=327) cohorts.

### Baseline features and clinical findings

Patient socio-demographic factors, comorbidities, and clinical findings are shown in Table 1. Male adults consisted of 47.2% (292/619) of total cases and were more likely to be younger, uninsured, and had more comorbidities and immunosuppressive conditions than females. Comorbidity as defined by a Charlson Comorbidity Index score >=1 was present in 32.2% of males vs. 22.3% of females (p=0.006). In addition, 18.2% of males vs. 9.2% of females were immunosuppressed (p=0.001), and 16.2% of males vs. 5.5% of females had HIV infection and AIDS (p =< 0.001). In regards to presenting symptoms or signs, females more often presented with nuchal rigidity (34.7% vs. 26.9%, p=0.045), while males were more often febrile (36.1% vs. 26.5%, p=0.010).

### Laboratory results and physician management

Laboratory results and patient follow-up information is show in Table 2. All patients received a lumbar puncture. Serum and CSF findings demonstrated no significant differences between males and females. Males were more likely to have a positive Gram stain (10.3% vs. 3.7%, p=0.003) and more often tested positive for *C. neoformans* than females (27.0% vs. 8.7%, p=<0.001). No other significant differences between the two groups were found in the microbiology analysis.

A head CT was performed on 89.3% of patients, of which males were more likely than females to have an abnormal result (10.1% vs. 4.9%, p=0.021). A brain MRI was performed on 46.8% of patients. Male patients were more likely to receive a brain MRI (51.7% vs. 42.5%, p=0.022), although no significant differences were found in the results of the scans between male and female cohorts. Follow up information was available at discharge on all patients. An adverse clinical outcome was found in 11.3% of patients, with no significant differences between cohorts.

### Etiologies and clinical outcomes

The majority of the patients had meningitis of an unknown etiology (407 patients, 65.8%) (Table 3). A diagnostic etiology was identified in 212 patients (34.2%). An urgent treatable etiology was most frequently diagnosed in males (26% vs. 15%, p<0.05) and this difference was driven by a higher proportion of cryptococcal meningitis cases in males (48% vs. 14%). Other urgent treatable etiologies included bacterial meningitis, herpes simplex encephalitis, *M. tuberculosis*, varicella zoster virus, and central nervous system lymphoma or carcinomatosis. A similar proportion of cases of untreatable etiologies (e.g., West Nile virus, enterovirus, St. Louis encephalitis virus, and Epstein-Barr virus), and nonurgent treatable etiologies (e.g., herpes simplex meningitis and acute human immunodeficiency virus) was seen in males and females. Females with an urgent treatable etiology had worse outcomes than males (40% vs. 21%, p<0.05). There were no differences in adverse clinical outcomes between males and females in the unknown, untreatable and nonurgent treatable etiologies.

### Factors associated with adverse clinical outcomes

On bivariate analysis, the variables significantly associated with an adverse clinical outcome in the male cohort (p<0.05) included: age greater than 60 years, abnormal neurological examination, fever (T >38.4°C) and abnormal laboratory findings (serum leukocyte ≥ 12,000 cells/µL, elevated CSF protein ≥ 100 mg/dL, and decreased CSF glucose <45 mg/dL) (Table 4). On logistic regression analysis, an abnormal neurological examination (odds ratio (OR): 18.61; 95% confidence interval (CI): 4.75–72.86), fever (OR: 7.16; 95% CI: 2.35–21.86), and a CSF glucose <45 mg/dl (CI: 5.07; 95% CI: 1.54–16.68) were validated by bootstrap analysis and still significantly associated with an adverse clinical outcome (p<0.05) (Table 5).

In the female cohort, we found that age greater than 60 years, Charlson Comorbidity Index score ≥ 1, abnormal neurological examination, fever, and abnormal laboratory findings (serum leukocyte ≥ 12,000 cells/µL, elevated CSF protein ≥ 100 mg/dL, and decreased CSF glucose < 45 mg/dL) were significant predictors of an adverse clinical outcome using bivariate analysis (Table 5). However, after logistic regression with bootstrap analysis, only age greater than 60 years of age (OR:6.22; 95% CI:2.60–14.90) and an abnormal neurological examination (OR:7.96; 95% CI:3.15–20.07) remained significant (p<0.05).

## Discussion

It is well established that the makeup of the female brain differs from the male, at morphological, neurochemical, and functional levels of organization [10]. In the realm of infectious disease, illnesses rarely affect males and females equally, largely due to sex-related physiology and partly due to gender-specific behavior [11]. However, gender differences in neurological diseases, particularly meningitis, are seldom investigated. This study is, to the best of our knowledge, the first and largest to analyze gender differences in clinical features of and prognostic factors for community-acquired meningitis.

This study demonstrated that community-acquired meningitis in male adults differs significantly from female adults in clinical presentation, etiologies, and outcomes. On clinical presentation, males more often presented with coexisting medical conditions, including immunosuppression, HIV/AIDS, and a Charlson Comorbidity Index score ≥ 1 (Table 1). HIV infection is more commonly seeing in males as they engage in more risky behavior as documented by studies done by the Center for Disease Control and Prevention (CDC) [12]. We also found that males were more often febrile, and this could be due possibly to a higher proportion of fever in HIV/AIDS patients (44.0% in HIV positive vs. 29.5% in HIV negative, p=0.016). Females presented more often with nuchal rigidity on clinical examination; the reason for this difference is unclear.

Both the male and female cohorts were managed similarly with no significant differences in rates of admission, empirical antibiotic or antiviral therapy, or head CT imaging (Table 2). However, male patients were more likely to have an abnormal CT scan, and this could probably be explained by the higher rates of abnormal head CT scans in HIV positive patients (16.3% vs. 6.3%, p=0.006). In addition, the abnormalities on head CT scan prompted further imaging, resulting in more brain MRIs performed on males than females. On microbiology analysis, males were more likely to test positive for *C. neoformans* and have a positive Gram stain. This higher incidence of *C. neoformans* in males is consistent with previous studies [13], and can be also be explained by the higher prevalence of HIV/AIDS in the male cohort, which causes immune system suppression. Cryptococcal disease is one of the most important opportunistic infections related to AIDS [13,14]. Furthermore, the greater percentage of positive Gram stains is due to a higher proportion of yeast found on the male cohort (Table 3).

Meningitis of an unknown cause accounted for 65.8% of total cases (Table 3). Unknown etiologies are a diagnostic challenge to physicians, as the main benefit in recognizing a treatable etiology is early administration of the appropriate therapy [15,16]. Of known causes, male patients were more likely to have urgent treatable etiologies due to a higher rate of *C. neoformans*. No difference was found between male and female patients in regards to untreatable and nonurgent treatable etiologies. Interestingly, female patients with urgent treatable etiologies were found to have worse outcomes than males. The reason for this is not readily apparent. However, literature has similarly shown disease outcome to be worse in females in cases of measles, toxoplasmosis, dengue, or hantavirus infections. This could be attributed to elevated humoral responses in women, leading to detrimental effects [17].

We found abnormal neurological examination, fever, and CSF glucose <45 mg/dL were poor prognostic factors in males, while age greater than 60 years and abnormal neurological examination were poor prognostic factors in females (Table 5). Neurological compromise was a strong predictor of adverse outcomes in both cohorts, which is consistent with previous research on bacterial meningitis in adults [4,18]. The reason for the differences in the other prognostic factors between genders is unknown.

Our study had several strengths. First, this is to our knowledge the only study evaluating gender differences in community-acquired meningitis. Secondly, the large sample size allowed us to perform valid statistical analysis using multivariable logistic regression with bootstrap analysis. Third, all baseline information was obtained at a defined time and used variables that are commonly available to clinicians. Lastly, we assessed the study endpoint with the well-validated Glasgow outcome scale that has been used in several meningitis studies.

Despite our strengths, the study also had some limitations. As a retrospective study design, the diagnostic examination could not be standardized for every patient, resulting in missing data. The patients enrolled in the study were drawn from hospitals in the Houston area, so the results should not be generalized to other areas without additional confirmatory studies. Lastly, the majority of the patients had meningitis of an unknown etiology (65.8%), which shows there is much to be understood about this disease. With further research into diagnostic guidelines, we hope to improve our understanding of this disease.

## Conclusion

Community-acquired meningitis in males differs significantly from females in regards to clinical presentations, laboratory and imaging analysis, and predictors of adverse clinical outcomes. Males more often present with coexisting medical conditions and show abnormalities in laboratory and imaging results, while females more often complain of stiff neck and present with nuchal rigidity. Female patients have worse outcomes for meningitis of urgent treatable etiologies. While fever, abnormal CSF glucose, and an abnormal neurological examination are significant predictors of an adverse clinical outcome in male patients, age greater than 60 years of age and an abnormal neurological examination are poor prognostic factors in female patients. Better diagnostic tools and guidelines are needed to explore and apply these gender differences to standardize disease management.

## Figures and Tables

**Table 1:** Baseline characteristics of adults with community-acquired meningitis according to gender (N=619).

Clinical Feature	Male, n=292	Female, n=327	P-value
Age, median (range)	36 (18–86)	38 (18–92)	0.044
**Race, N (%)**			
Caucasian	124 (42.5)	163 (49.8)	0.066
African American	83 (28.4)	85 (26.0)	0.497
Hispanic	76 (26.0)	70 (21.4)	0.176
Other	9 (3.1)	9 (2.8)	
Uninsured, n/N (%)	107/290 (36.9)	83/327 (25.4)	0.002
**Coexisting medical conditions, n/N (%)**			
Charlson Comorbidity Index score ≥ 1	94/292 (32.2)	73/327 (22.3)	0.006
Immunosuppressed^a^	53/292 (18.2)	30/327 (9.2)	0.001
HIV/AIDS	47/291 (16.2)	18/326 (5.5)	<0.001
History of injection drug use	8/287 (2.8)	4/325 (1.2)	0.166
Sinusitis or otitis	21/292 (7.2)	20/327 (6.1)	0.591
**Presenting symptoms, n/N (%)**			
Headache	253/281 (90.0)	294/318 (92.5)	0.294
Nausea	178/274 (65.0)	225/319 (70.5)	0.147
Subjective fever	189/286 (66.1)	197/325 (60.6)	0.162
Stiff neck	106/274 (38.7)	159/313 (50.8)	0.003
Photophobia	96/250 (38.4)	122/291 (41.9)	0.405
Malaise	105/276 (38.0)	118/311 (37.9)	0.98
Respiratory symptoms	36/276 (13.0)	36/319 (11.3)	0.512
**Presenting signs, n/N (%)**			
Nuchal rigidity	70/260 (26.9)	109/314 (34.7)	0.045
Temperature >38.4°C	104/288 (36.1)	86/325 (26.5)	0.01
Abnormal neurological examination^b^	91/292 (31.2)	81/327 (24.8)	0.076
Vesicular or petechial rash	5/285 (1.8)	6/324 (1.9)	0.928

a Individuals with HIV, AIDS, organ transplants, congenital diseases, steroid use and other immunocompromising conditions;

b Seizure, abnormal mental status (disorientation or Glasgow Coma Scale <15), cranial nerve abnormality, focal motor deficit, or aphasia.

**Table 2:** Laboratory Results and Follow-up of Adults with Community-Acquired Meningitis According to Gender (N=619).

Clinical Feature	Male, n=292	Female, n=327	P-value
**Blood and CSF analysis**			
Serum leukocyte count, cells/uL, median (range)	8,400 (900 – 34,800)	8,900 (1,400 – 43,500)	0.813
CSF leukocyte count, cells/uL, median (range)	104 (6 – 53,600)	200 (40 – 40,064)	0.947
CSF protein, mg/dL, median (range)	82 (18 – 659)	82 (22 – 706)	0.579
CSF glucose, mg/dL, median (range)	57 (1 – 421)	55 (1 – 366)	0.222
Serum leukocyte ≥ 12,000 cells/uL, n (%)	72 (24.7)	80 (24.5)	0.956
CSF protein ≥ 100 mg/dL, n (%)	116 (39.7)	121 (37.0)	0.487
CSF glucose <45 mg/dL, n (%)	53 (18.2)	63 (19.3)	0.723
Microbiology analysis, n/N (%)			
Positive Gram stain	30/291 (10.3)	12/327 (3.7)	0.003
Positive blood culture	186/292 (63.7)	214/327 (65.4)	0.563
Positive polymerase chain reaction test^a^	34/292 (22.8)	39/327(27.1)	0.399
**Positive CSF culture**			
Bacterial	20/280 (7.1)	14/325 (4.3)	0.131
Viral	0/69 (0.0)	2/60 (3.3)	0.126
*C. neoformans*^b^	33/122 (27.0)	8/92 (8.7)	<0.001
**Management decision, n/N (%)**			
Admitted to hospital	284/292 (97.3)	317/327 (96.9)	0.814
Empirical antibiotic therapy	212/288 (73.6)	240/323 (74.3)	0.846
Empirical viral therapy	75/291 (25.8)	82/325 (25.2)	0.877
Head computed tomography performed	268/292 (91.8)	285/327 (87.2)	0.063
Abnormal^c^	27/268 (10.1)	14/285 (4.9)	0.021
Brain magnetic resonance imaging performed	151/292 (51.7)	139/327 (42.5)	0.022
Abnormal^d^	58/151 (38.4)	51/139 (36.7)	0.763
Adverse clinical outcome	29/292 (9.9)	41/327 (12.5)	0.307

a Includes HSV, VZV, and enterovirus.

b Positive fungal culture or cryptococcal antigen.

c Focal or nonfocal intracranial abnormalities.

d Mass lesions, strokes, hypoattenuations, meningeal enhancement, bleeds, white matter abnormalities.

e Glasgow Outcome Scale score of <4.

**Table 3:** Etiologies and Adverse Outcomes (AO) in Individuals with Community-Acquired Meningitis According to Gender (N=619).

Etiology	Male, n=292		Female, n=327	
	Participants	AOs	Participants	AOs
Unknown, n (%)^a^	175 (60)	8 (5)	232 (71)	12 (5)
Urgent treatable, n (%)^a^,^b^	77 (26)	16 (21)	50 (15)	20(40)
Bacterial meningitis, n^c^	23		23	
*C. neoformans*, n	37		7	
Herpes simplex encephalitis, n	3		5	
*M. tuberculosis*, n	3		2	
Varicella zoster virus, n	2		5	
Central nervous system lymphoma or carcinomatosis	0		2	
Other^d^	3		3	
Untreatable, n (%)	22 (8)	5 (23)	22 (7)	9 (41)
West Nile virus	15		14	
Enterovirus	5		6	
St. Louis encephalitis virus	1		2	
Epstein-Barr virus	1		0	
Nonurgent treatable, n (%)	18 (6)	0 (0)	23 (7)	0 (0)
Herpes simplex meningitis	17		22	
Acute human immunodeficiency virus	2		1	
Other^e^	2		2	
Total, n (%)	292 (100)	29 (10)	327	41(13)

a P <0.05 comparing the etiologiesa and ACOs.

b between the male and female cohorts.

c Organisms identified include *S. pneumoniae, E. cloacae, Enterococcus, H. influenzae, L. monocytogenes*, Methicillin-sensitive *S. aureus, N. meningitides, S. aureus*, coagulase-negative Staphylococcus, Group A Streptococcus, Group B Streptococcus, *S. anginosus milleri*.

d Other urgent treatable etiologies include systemic lupus, toxoplasmosis, histoplasmosis, *cerebral aneurysm, Brucella*, and *E. coli* UTI.

e Other nonurgent treatable etiologies include multiple sclerosis, neurosyphilis, influenza virus type A, and CMV.

**Table 4:** Factors Associated with an Adverse Outcome in Adults with Community-Acquired Meningitis According to Gender (N=619) on Bivariate Analysis.

Characteristic	Male, n = 292	Female, n = 327
	Odds Ratio (95% Confidence Interval) P-Value	Odds Ratio (95% Confidence Interval) P-Value
Age > 60 years	0.36 (0.17–0.77) 0.010	0.12 (0.067–0.21) <0.001
Baseline characteristics		
Charlson Comorbidity Index score ≥ 1	0.58 (0.29–1.16) 0.125	0.225 (0.13–0.39) <0.001
Immunosuppressed	0.85 (0.36–1.98) 0.709	0.727 (0.31–1.71) 0.474
Sinusitis or otitis	0.48 (0.19–1.26) 0.147	0.47 (0.21–1.06) 0.082
Presenting features		
Abnormal neurological examination^a^	0.05 (0.02–0.17) <0.001	0.09 (0.05–0.19) <0.001
Temperature >38.4°C	0.19 (0.08–0.43) <0.001	0.51 (0.29–0.90) 0.020
Laboratory Findings		
Serum white blood cell count ≥ 12,000 cells/uL	0.31 (0.15–0.60) <0.001	0.457 (0.26–0.81) 0.007
CSF protein ≥ 100 mg/dL	0.21 (0.09–0.48) <0.001	0.376 (0.21–0.68) 0.001
CSF glucose <45 mg/dL	0.36 (0.18–0.72) 0.004	0.460 (0.26–0.83) 0.010

a Seizure, abnormal mental status (disorientation or Glasgow Coma Scale <15), cranial nerve abnormality, focal motor deficit, or aphasia.

**Table 5:** Logistic Regression Analysis of Factors Independently Associated with Adverse Outcome in Adults with Community-Acquired Meningitis (N=619).

Characteristic	Male, n=292	Female, n=327
	Odds Ratio (95% Confidence Interval)	Odds Ratio (95% Confidence Interval)
Age >60 years		6.22 (2.59–14.90)
Presenting Features		
Abnormal neurological examination^b^	18.61 (4.75–72.86)	7.96 (3.15–20.07)
Temperature >38.4 °C	7.16 (2.35–21.86)	
Laboratory Findings		
CSF glucose <45 mg/dL	5.07 (1.54–16.68)	

b Seizure, abnormal mental status (disorientation or Glasgow Coma Scale <15), cranial nerve abnormality, focal motor deficit, or aphasia.
